# In vivo visualization and characterization of inflamed intestinal wall: the exploration of targeted microbubbles in assessing NF‐κB expression

**DOI:** 10.1111/jcmm.16858

**Published:** 2021-08-19

**Authors:** Chenyang Zhao, Li Ma, Yanwen Luo, Wenbo Li, Mengsu Xiao, Qingli Zhu, Yuxin Jiang

**Affiliations:** ^1^ Department of Ultrasound Chinese Academy of Medical Sciences and Peking Union Medical College Hospital Chinese Academy of Medical Sciences and Peking Union Medical College Beijing China

**Keywords:** contrast‐enhanced ultrasound, inflammatory bowel diseases, microbubbles, molecular imaging, NF‐κB

## Abstract

NF‐κB, a critical cytokine of inflammatory bowel diseases (IBD), is a viable marker to reflect the inflammatory activity of the intestine. We aimed to develop NF‐κB‐targeted microbubbles (MBs) and perform molecular contrast‐enhanced ultrasound (CEUS) to quantify NF‐κB expressions on the intestinal wall in IBD mice in vivo. In this study, NF‐κB‐targeted MBs were fabricated by connecting biotin‐loaded NF‐κB antibodies and avidin‐loaded MBs. NF‐κB‐targeted MBs presented as transparent and round bubbles with an average diameter of 1.03/μm±0.01. The specific binding of targeted MBs and inflammatory cells was validated by in vitro experiments, including flow cytometry, Western blot and immunofluorescence, which revealed the specific binding of targeted MBs and inflammatory cells. Subsequently, NF‐κB‐targeted CEUS imaging was performed on mice with chemical‐induced colitis, and the peak intensity (PI) and time‐to‐peak (TTP) were quantified. Pathological and immunohistochemical (IHC) examinations were further implemented. For the target CEUS group, fast enhancement followed by slow subsiding was observed. The PI of target CEUS of the IBD mice was significantly higher than that of non‐target CEUS of the IBD mice, healthy controls and target CEUS of the treated IBD mice (34835%[13379–73492%] VS 437%[236–901%], 130%[79–231%], 528%[274–779%], ***p***<0.0001), in accordance with the IHC results of NF‐κB expressions. The TTP of target CEUS of the treated mice was significantly higher than that of untreated mice (35.7s [18.1–49.5s] VS 8.3s [4.2–12.5s], *p*<0.0001). Therefore, we suggested that NF‐κB‐targeted CEUS could accurately detect and quantify NF‐κB expressions on the intestinal walls of IBD, enabling the evaluation of intestinal inflammation.

## 1 INTRODUCTION

Inflammatory bowel diseases (IBD) are systematic, chronic inflammatory diseases, including ulcerative colitis (UC) and Crohn disease (CD).^1^, ^2^ The disease often begins in adolescence and has a high recurrence rate, causing a huge economic and social burden. Chronic inflammation resulting in mucosal damage is essential to the pathogenesis of IBD.^3^, ^4^ The NF‐κB pathway has been proved to be highly involved in the chronic inflammation process of IBD.^5^ NF‐κB is an essential family of inducible transcription factors that is intensively involved in the inflammatory processes and immune responses, including p50 (NF‐κB1) and p52 (NF‐κB2), p65 (RelA), RelB and c‐Rel (Rel). The activity of NF‐κB is triggered by the TNF family through different signalling pathways.^6^ NF‐κB family has alsso been proved to present a high‐level expression in the intestinal mucosa of IBD and play a vital role in the mucosal healing of IBD.^7^, ^8^ Consequently, NF‐κB has been deemed as an essential target of diagnosis and treatment of the disease.^9^, ^10^ A variety of natural and novel synthetic agents are developed to ameliorate colitis by targeting NF‐κB, in addition to conventional anti‐inflammatory agents.^11^ Studies have shown that the expression level of NF‐κB on intestinal epithelial cells is not only highly associated with the disease activity, but also indicative of the treatment response and the risk of developing colitis‐associated cancer.^12^, ^13^, ^14^ Hence, an accurate assessment of NF‐κB expressions in vivo can be helpful in guiding the management of IBD.

Molecular imaging is an ideal method for in vivo detection and characterization of NF‐κB expressions on the inflamed intestinal wall. The utilization of contrast‐enhanced ultrasound (CEUS) in molecular imaging has been explored recently. Microbubbles (MBs), the contrast agents of CEUS, can be coupled with specific molecules to facilitate in vivo molecular CEUS. When inflammatory markers bind to MBs, a noninvasive detection and quantification of overexpressed inflammatory molecules can be realized via CEUS. To note, due to its noninvasiveness and convenience, bowel ultrasound (US) has been recommended as a conventional imaging method for IBD in clinical practice.^15^, ^16^ Hence, the application of molecular CEUS in evaluating IBD can be promising in clinical practice. Previous studies have proved the feasibility of target CEUS in evaluating murine colitis by depicting the expressions of inflammatory molecules, including P‐selectin and mucosal addressin cellular adhesion molecule (MAdCAM)‐1.^17^, ^18^ Therefore, it is reasonable to assume that CEUS can also be used to evaluate the NF‐κB expression in intestinal epithelial cells in vivo, thus facilitating further disease management.

In this study, we fabricated NF‐κB‐p65‐targeted MBs by decorating NF‐κB‐p65 antibody molecules onto the surface of MBs, and NF‐κB‐targeted CEUS was performed on murine models with chemically induced colitis to quantify NF‐κB‐p65 expressions of colon tissues, aiming to explore its potentials in the evaluation and treatment monitoring of IBD.

## 2 MATERIALS AND METHODS

### 2.1 Fabrication and characterization of NF‐κB‐targeted MBs

Avidin‐loaded microbubbles (Labeler LS, 0.8ml liquid containing 1x10^10^ of MBs) and naked MBs (Prime, 0.8ml liquid containing 1x10^10^ of MBs) were synthesized by Boxin Biotechnology Inc (Taipei, Taiwan). Fluorescein isothiocyanate (FITC)‐biotin double‐labelled NF‐κB‐p65 Abs (Abcam, purchased from Aobsen Biochem Co., Ltd., Shanghai, China) were synthesized, and 50μg of the double‐labelled Abs was mixed with Avidin‐MBs for 30 minutes at the room temperature. Before mixing, the Avidin‐MBs were well shaken. Then, the mixture was centrifugated at 1500rmp/min for 5 minutes. Unbinding Abs were removed after washing with PBS solution. The upper layer of the mixture was obtained and subjected to volume measurement. In this way, the NF‐κB‐p65‐targeted MBs were fabricated. The solution of targeted MBs was put in a counting plate to count the targeted MBs and measure the particle sizes (Multisizer 4e, Beckman, USA). We also observed the targeted MBs under a microscope (BX53, Olympus, Tokyo, Japan).

### 2.2 The detection of binding rate of NF‐κB‐Abs and MBs

A total of 400μl NF‐κB‐p65‐targeted MBs loaded with different volumes of NF‐κB‐p65 Abs (5μg, 10μg, 40μg) were used for detection of the binding rate using flow cytometry of Annexin V‐FITC /propidium iodide (PI) double staining (Fluorescence microscopy: Olympus, BX53, Japan; flow cytometer: BD Biosciences FACSCanto II Flow Cytometer [Becton Dickinson, San Jose, CA, USA]). Avidin‐MBs loaded with IgG‐Abs were also prepared as the control. The groups listed as follows were assessed by fluorescence microscopy (BX53, Olympus, Tokyo, Japan). Group 1: naked MBs; Group 2: NF‐κB‐targeted MBs (5μg NF‐κB‐Abs); Group 3: NF‐κB‐targeted MBs (10μg NF‐κB‐Abs); Group 4: NF‐κB‐targeted MBs (40μg NF‐κB‐Abs); Group 5: avidin‐MBs loaded with 40μg IgG‐Abs.

### 2.3 Cellular uptake of MBs in vitro

#### 2.3.1 Cell culture and grouping

The murine macrophage cell line, RAW 264.7, was used for cellular uptake assay, and murine embryonic fibroblast, NIH 3T3, was used as the control. The cell lines were purchased from the National Infrastructure of Cell Line Resource (Shanghai, China). The cells were cultured in Dulbecco's modified eagle medium (DMEM) (Welgene, Daegu, Korea) and treated with 10% foetal bovine serum (Welgene, Daegu, Korea) under 5% of CO_2_ and the temperature of 37℃. The cells were digested with 0.25% trypsin for passage every 2–3 days. The cells in logarithmic growth phase were used after being washing by phosphate buffer saline (PBS) (Origene Biotechnology Co., Ltd, Wuxi, China) for subsequent experiments. Before being mixed with MBs, RAW 264.7 cells were seeded in 6‐well plate and cultured with lipopolysaccharide (LPS, 100 ng/ml) (Sigma‐Aldrich Biotechnology Co., Ltd, Shanghai, China) for 5h.

The following groups were set for flow cytometry, Western blot and immunofluorescence: Group 1: saline +NIH 3T3; Group 2: saline +RAW267.4; Group 3: Naked MBs +NIH 3T3; Group 4: Naked MBs +RAW264.7; Group 5: FITC‐NF‐κB‐targeted MBs +NIH 3T3; Group 6: FITC‐NF‐κB‐targeted MBs +RAW264.7.

#### 2.3.2 Flow cytometry

The cells were collected in a 1.5mL‐EP tube and added with 200ul of the MBs according to the grouping method. After incubating for 20 minutes, the volume of the mixture was made up to 400μL by adding PBS. Flow cytometry (Annexin V‐FITC/PI double staining) was performed subsequently for detecting the binding status of MBs and the cells.

#### 2.3.3 Western blot

The cells were collected in a 1.5mL‐EP tube and added with 200ul of the MBs according to the grouping method. After incubating for 20 minutes, the cells were collected and centrifugated. The Western blot experiment was performed with reference to the protocol proposed by Thomas S. Hnasko et al.^19^ The harvested cells were treated with cold lysis buffer. The protein concentration was then measured using bicinchoninic acid (BCA) assay kit (Sigma‐Aldrich Biotechnology Co., Ltd, Shanghai, China). The protein sample was diluted by sodium dodecyl sulphate (SDS) buffer concentrate and heated for 10min between 70–100℃. Then, the samples were loaded into the gel wells and underwent polyacrylamide gel electrophoresis (PAGE) for migration and separation (Power Supplies Basic, Bio‐Rad, USA). The separated samples were transferred to polyvinylidene fluoride (PVDF) membrane and immobilized for further immunodetection. After being washed and blocked in 5% skim milk for 4 h, the membranes with samples were then incubated with rabbit anti‐NF‐κB‐p65 antibodies and goat anti‐rabbit IgG/ horseradish peroxidase [HRP] antibodies (Abcam, ab16502, purchased from Aobsen Biochem Co., Ltd., Shanghai, China). Glyceraldehyde‐3‐phosphate dehydrogenase (GAPDH) (Abcam, ab181602, Aobsen Biochem Co., Ltd., Shanghai, China) was used as the loading control. A blotting detection system was used to observe the blots (SYNGENE G:BOXChemiXR5, Eppendorf, Germany). A gel documentation system (Gel‐Pro32, Bio‐Rad, Hercules, CA, USA) was used for the quantitative evaluation of immunoblotting.

#### 2.3.4 Immunofluorescence

The cells were collected and added with the MBs according to the above groupings. After incubated at room temperature for 30 minutes, the mixture was added with dihydrochloride (DAPI) (Origene Biotechnology Co., Ltd, Wuxi, China) staining solution. Then, it was washed with PBS for 3 times and put on the glass side for observing under the fluorescence microscope. The fluorescence microscope shooting for each group was performed under the same field of view and magnification. And the results of three different fields of view under the same magnification for each group were obtained. The binding rate was evaluated through the intensity and area of fluorescence under microscope, defined as ‐, +, ++, +++.

#### 2.3.5 Confocal laser scanning

We further performed confocal laser scanning to observe the intracellular distributions of the NF‐κB‐targeted MBs. The cells were added with NF‐κB‐FITC‐targeted MBs and incubated for 30 min. Then we used DAPI fluorescent dyes to stain the nuclei of cells. The confocal laser scanning microscopy (Nikon Ti‐E+C2, Japan) was performed to observe the intracellular distributions of the FITC‐NF‐κB‐targeted MBs. For DAPI microscopic imaging, the excitation wavelength was 358 nm, and the emission wavelength was 461 nm. For FITC on the NF‐κB microscopic imaging, the excitation wavelength was 495 nm, and the emission wavelength was 520 nm.

### 2.4 In vivo CEUS imaging of animal model

#### 2.4.1 DSS‐induced colitis model and grouping

The animal experiment was approved by the Institutional Animal Care and Use Committee of Peking Union Medical College Hospital. A total of 30 female C57BL/6 mice (6–8 weeks of age, 18‐24g) (Beijing, China) were prepared and acclimatized for 2 weeks before modelling at a room temperature. Twenty‐four mice were distributed to the model groups and were fed with 3% dextran sodium sulphate (DSS) (Thermo Fisher, Shanghai, China) solution for nine days. Six mice were fed with normal drinking water for nine days as the control.

Twelve mice of the 24 modelling mice were used as the treatment group. On Day 9, the DSS solution was switched to drinking water. And six mice were administrated with Mesalazine Enemas (Dr. Folk Pharma GmbH, Freiburg, Germany) through enema for 3 consecutive days (Mesalazine Enemas solvent, 0.1ml per mouse, once per day, 3 days in total). Another six mice in the experimental group were used as the non‐treatment group. After drinking DSS water on Day 9, they switched to ordinary drinking water and were treated with 0.1ml saline for 3 consecutive days (0.1ml per mouse, once a day, 3 days in total).

The mice were divided into five groups, with 6 mice in each group, listed as follows:

Group A: NF‐κB‐targeted CEUS +IBD mice with colitis on Day 9; Group B: non‐target CEUS +IBD mice with colitis on Day 9; Group C: NF‐κB‐targeted CEUS +healthy mice on Day 9; Group D: NF‐κB‐targeted CEUS +Mesalazine‐treated IBD mice on Day 12; Group E: NF‐κB‐targeted CEUS +untreated IBD mice on Day 12.

#### 2.4.2 Clinical assessment

The clinical assessments of the mice were implemented on Days 0, 6, 9 and 12, including weight measurement and stool evaluation.

#### 2.4.3 In vivo CEUS imaging of IBD animal model

CEUS was performed using an ultrasonic machine, Zonare (Mindray, Shenzhen, China), with a high‐frequency linear probe (18‐30MHz, centred at 20MHz). After anaesthetizing (chloral hydrate, 3%, 0.1ml/mice) and shaving the mice, the mice were fixed onto a heated plain (37–40℃). The linear probe was put on the belly of the mice. And a gel pad was placed between the animal belly and the ultrasonic probe to remove the air. Grey‐scale US was performed to find the target location for CEUS. Based on the anatomical features of the colon and previous studies, we selected the bladder and kidney poles as anatomical landmarks to find the target site of the colon.^4^, ^20^ Firstly, we found the filled bladder which presented as anechoic elliptical structure in the bottom of the belly. The sigmoid colon usually passed next to the bladder. If the bladder was empty after anaesthesia, we would select the colon segment near the lower pole of the right or left kidney. The selected colon segment was put into the centre of the screen as the target for CEUS. After localization, the US mode was switched to the harmonic CEUS, and a bolus of 50μL MBs (concentration: 0.8ml liquid containing 1x10^10^ of MBs) was injected intravenously via murine tail vein. The CEUS imaging lasted for 90 seconds. The imaging depth was 2cm, and other imaging settings were also kept unchanged during the process. For each mouse, only one injection of MBs was done and CEUS was observed at only one site. Group A‐C received the imaging examination on Day 9 and Group D‐E on Day 12.

#### 2.4.4 Quantification of CEUS parameters

The SonoLiver Software (Philips, Andover, USA) for quantitative evaluation of CEUS was applied to delineate the time‐intensity curves (TICs) and calculate the CEUS‐related parameters, including time‐to‐peak (TTP) and peak intensity (PI) of the regions of interest (ROIs). For each animal, a segment of colon wall with 0.5cm length was selected as the ROI. After determining the ROI, the calculation would be done by the software automatically. For each CEUS examination, we performed three times of ROI drawing for calculation on the same bowel segment and took the mean value. The mean values of the parameters of different groups were utilized for analysis.

#### 2.4.5 Pathological and immunohistochemical (IHC) examination

Groups A‐C were sacrificed on Day 9, and Groups D‐E were sacrificed on Day 12 after treatment. The colons were removed and embedded in OCT compound. Colon tissues with 5μm of thickness, resected from the sections next to the bladder, or at the left or right kidney pole, were received hematoxylin and eosin (HE) stain. The histological changes of the samples were evaluated under microscopy. Colon tissues were fixed and blocked before the three‐step staining of IHC. The IHC examination was performed according to the protocol presented by Mori H et al.^21^ The samples were incubated with rabbit anti‐NF‐κB‐p65 antibodies, biotinylated goat anti‐rabbit IgG and Streptavidin Alexa Flour 546 conjugate (Abcam, ab16502, Aobsen biochem, Shanghai, China). Nuclei were counterstained with 4,6­diamidine­ 2­phenylindoldihydrochloride. The stained cells were counted and semi‐quantified under five HPF per section (average optical density [AOD] =integrated optical density [IOD] /area).

### 2.5 Statistical analysis

The statistical analysis was performed using IBM SPSS Statistics for Windows, Version 20.0 (IBM Corp, Armonk, NY, USA). The quantitative CEUS and pathological parameters were demonstrated as continuous variables (mean ±SD). Kruskal‐Wallis test and Student's t test was used to make comparisons among the groups. A p value of <0.05 was considered as significant.

## 3 RESULTS

### 3.1 Characterizations of the NF‐κB‐p65‐targeted microbubbles

**FIGURE 1:**
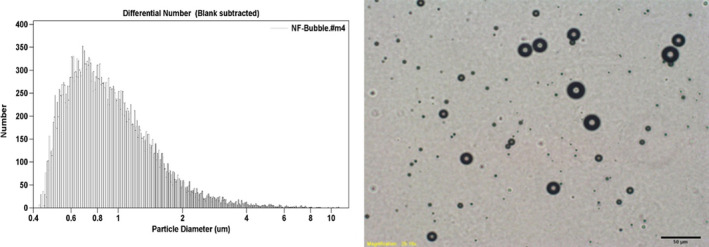
The distribution histogram illustrating the particle size and the microscope imaging of targeted MBs in white light. The MBs are presented as translucent oval bubbles (Magnification: ×400)

The targeted MBs had an average diameter of 1.03/μm±0.01 and a concentration of 1.5×10^9^/ml. The histogram illustrating the particle size distribution and the microscope image is shown in Figure 1.

### 3.2 The binding of NF‐κB‐p65 Abs and MBs

**FIGURE 2:**
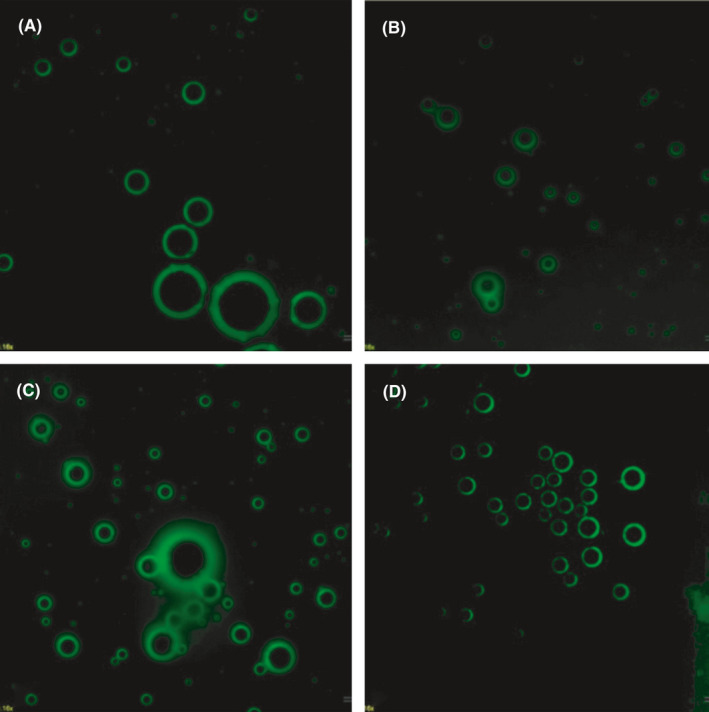
Results of fluorescence imaging showing the binding of fluorescein isothiocyanate (FITC)‐biotin double‐labelled antibodies and avidin‐MBs (Magnification: ×400). (A)5μg NF‐κB antibodies +400μL MBs; (B) 10μg NF‐κB + 400μL MBs; (C) 40μg NF‐κB + 400μL MBs; (D) 40μg IgG +400μL MBs. Under fluorescence microscopy, compact globular stereo‐structures with a uniform green aura were observed in each group, for which the MBs were treated with different concentrations of biotin‐FITC double‐labelled NF‐κB antibodies and IgG antibodies, indicating that the MBs could be successfully combined with the biotin‐FITC double‐labelled antibodies

**FIGURE 3:**
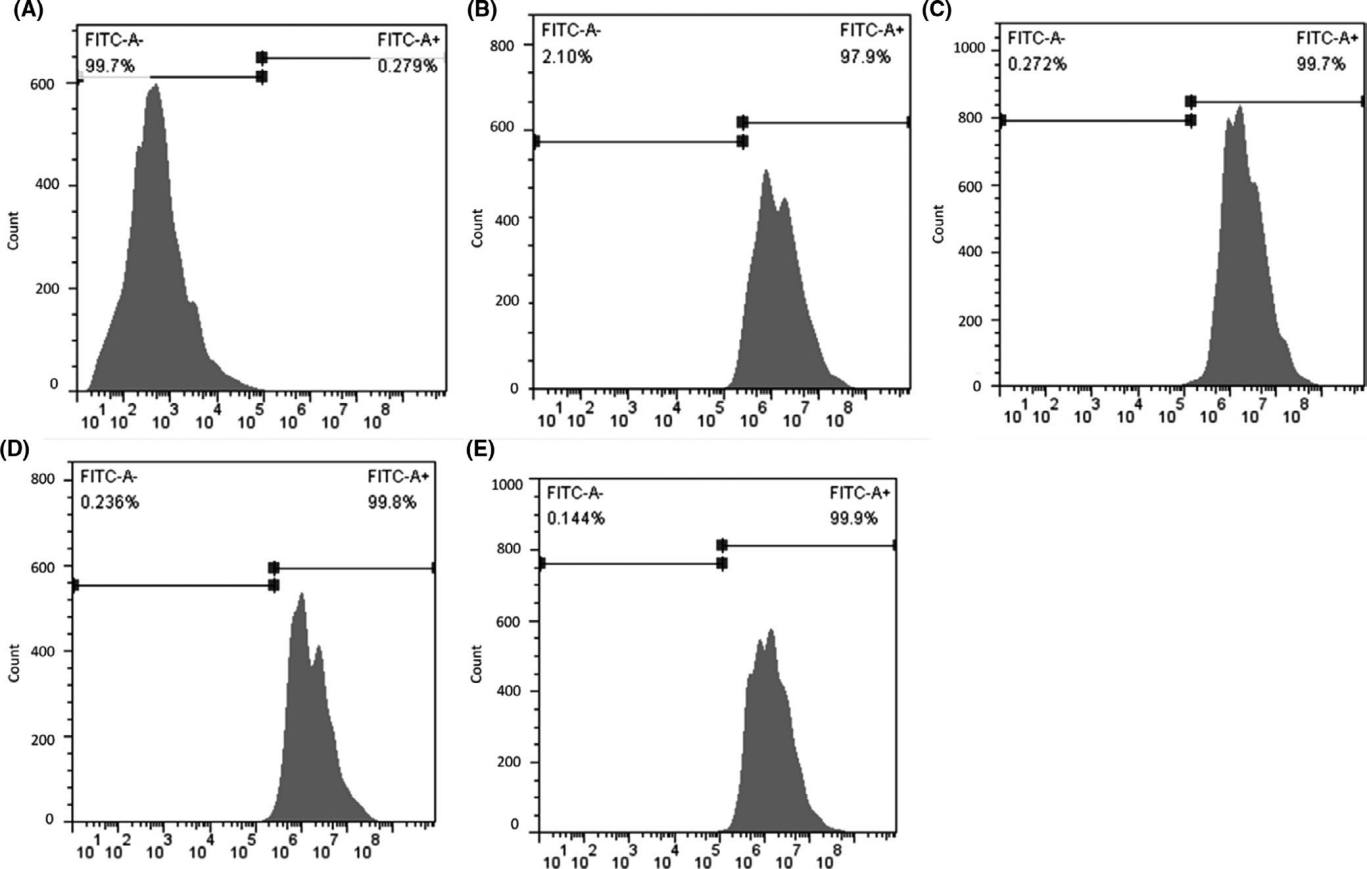
Results of flow cytometry: the binding rates of fluorescein isothiocyanate (FITC)‐biotin double‐labelled antibodies and avidin‐microbubbles (MBs) (X‐axis: fluorescence intensity, Y‐axis: count of MBs). (A) FL of naked MBs; (B) 5μg NF‐κB‐p65 antibodies +400μL MBs; (C) 10μg NF‐κB‐p65 antibodies +400μL MBs; (D) 40μg NF‐κB‐p65 antibodies +400μL MBs; (E) 40μg IgG antibodies +400μL MBs. After being treated with antibodies, high intensity of fluorescence could be detected in the MBs, which reached the saturation with 40μg antibodies

The MBs with different concentrations were successfully conjugated with biotin‐FITC double‐labelled NF‐κB‐p65 antibodies, depicted as compact globular stereo‐structures with a uniform green aura by fluorescence microscopy (Figure 2). The morphology and concentration remained unchanged after 30 minutes of incubation. According to the results of flow cytometry (Figure 3), the biotin‐FITC double‐labelled NF‐κB‐p65 antibodies with different concentrations and IgG antibodies specifically bond to avidin‐labelled MBs, and the binding rate reached saturation at 40μg of antibodies.

### 3.3 Cellular uptake of targeted MBs in vitro

**FIGURE 4:**
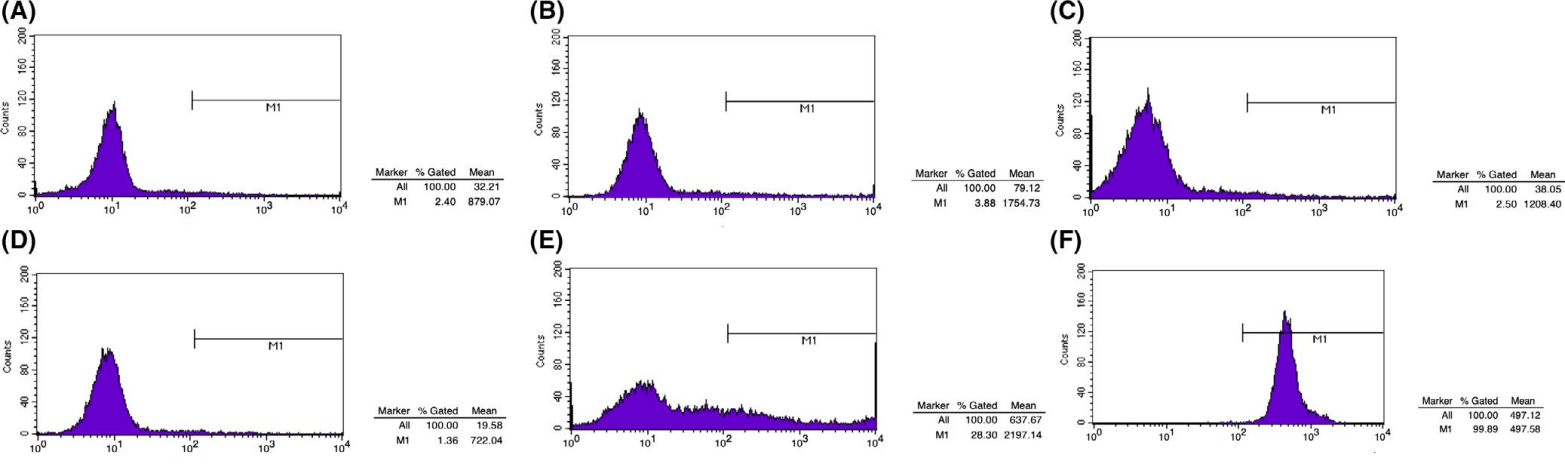
Results of flow cytometry: the binding rates of FITC‐NF‐κB‐targeted MBs and cells (X‐axis: fluorescence intensity, Y‐axis: count of cells). G1: saline +NIH 3T3; G2: saline +RAW267.4; G3: Naked MBs +NIH 3T3; G4: Naked MBs +RAW264.7; G5: FITC‐NF‐κB‐targeted MBs +NIH 3T3; G6: FITC‐NF‐κB‐targeted MBs +RAW264.7. In Group 5, 28% of the cells presented the fluorescence intensity of higher than 10^2^; and for Group 6, 99.9% of the cells presented the fluorescence intensity of higher than 10^2^, indicating that the target MBs could be specifically bond with RAW267.4 cells

**FIGURE 5:**
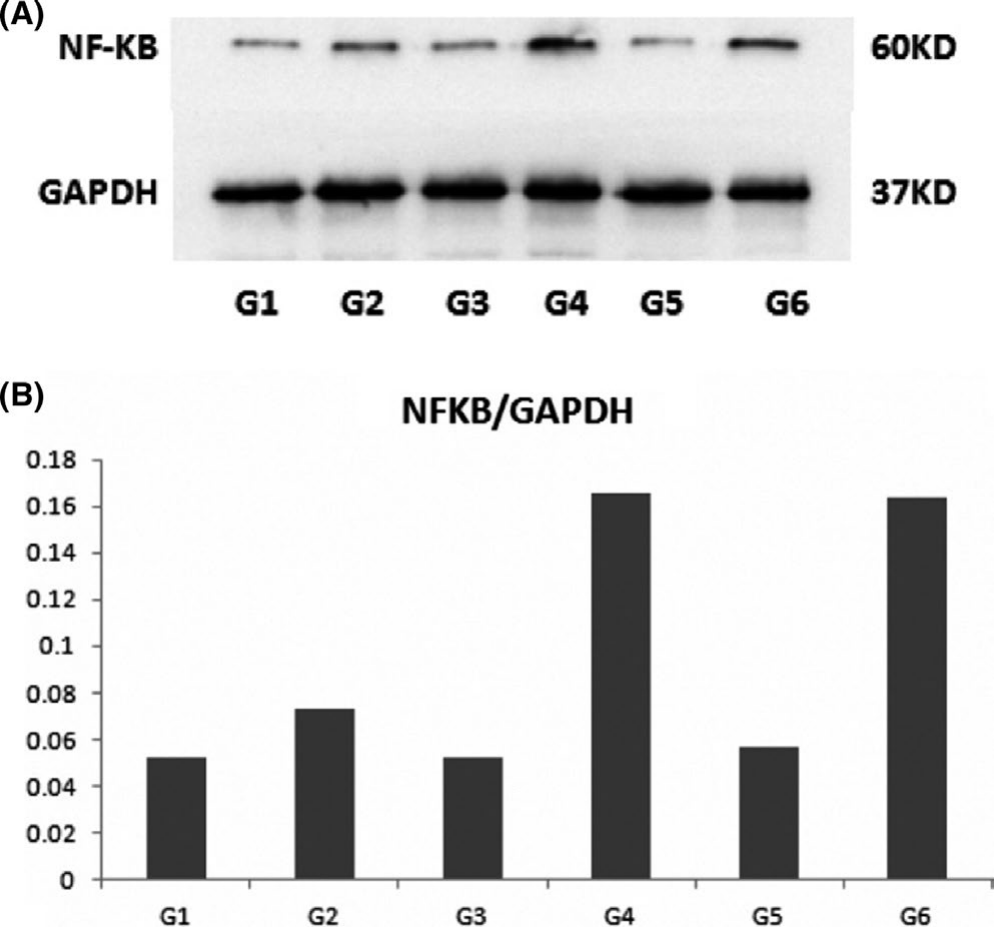
Results of Western blot. A. Blot images of the six groups; B. bar chart of blot analysis showing the expression level of NF‐κB. G4 and G6 had the highest expression level of NF‐κB‐p65. G1: saline +NIH 3T3; G2: saline +RAW267.4; G3: Naked MBs +NIH 3T3; G4: Naked MBs +RAW264.7; G5: FITC‐NF‐κB‐targeted MBs +NIH 3T3; G6: FITC‐NF‐κB‐targeted MBs +RAW264.7

**FIGURE 6:**
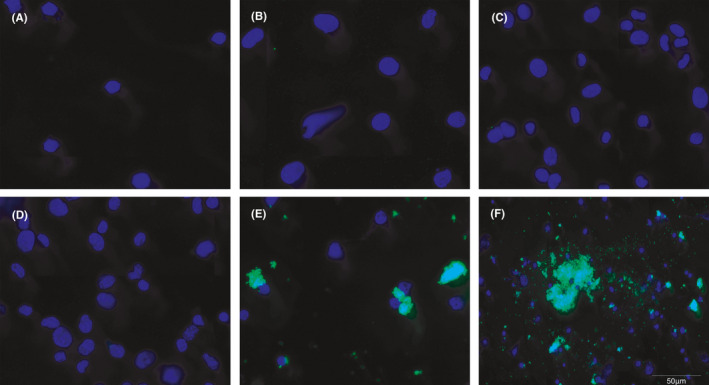
Results of immunofluorescence in vitro (Magnification: ×400). A. G1: saline +NIH 3T3; B. G2: saline +RAW267.4; C. G3: Naked MBs +NIH 3T3; D. G4: Naked MBs +RAW264.7; E. G5: FITC‐NF‐κB‐targeted MBs +NIH 3T3; F. G6: FITC‐NF‐κB‐targeted MBs +RAW264.7 Group 6 presented the strongest green fluorescence. A small range of green fluorescence was also observed in G5

The results of in vitro experiments are shown in Table 1 and Figures 4, 5, 6.

**TABLE 1:** The results of in vitro experiments.

Group No.		Flow cytometry FITC+	Western blot Grey value ratio of NF‐κB/GAPDH	Immunofluorescence
1	NIH 3T3	2.40	0.0522	‐
2	RAW264.7	3.88	0.0734	‐
3	Naked MBs +NIH 3T3	2.50	0.0524	‐
4	Naked MBs +RAW264.7	1.36	0.166	‐
5	FITC‐NFκB‐targeted MBs +NIH 3T3	28.30	0.0570	+
6	FITC‐NFκB‐targeted MBs +RAW264.7	99.89	0.164	+++

#### 3.3.1 Flow cytometry

According to the results of flow cytometry (Figure 4), Group 5 and 6 showed higher level of FITC+than the other four groups, indicating that the target MBs loading FITC and NFκB‐p65 antibodies were specifically conjugated with NIH 3T3 and RAW264.7 (FITC+of NIH 3T3: 28.30%, FITC+of RAW264.7: 99.89%).

#### 3.3.2 Western blot

The image of protein charts of Western blot examination and the grey value analysis histogram is illustrated in Figure 5, from which an enhanced expression of NF‐κB‐p65 could be observed in G4 and G6, indicating that the RAW264.7 cells had higher expression level of NF‐κB than NIH 3T3 cells.

#### 3.3.3 Immunofluorescence

The immunofluorescence images of the six groups are shown in Figure 6. Group 6 (FITC‐loaded NF‐κB‐targeted MBs +RAW264.7 cells) exhibited the strongest green fluorescence, which presented no difference before and after washing. This result validated the specific combination of target MBs and RAW264.7 cells. A small range of green fluorescence was also observed in G5, due to the relatively weak combination of target MBs and NIH 3T3 cells, in which inflammatory cytokines might also be expressed as a result of external stimulus.

#### 3.3.4 Confocal laser scanning

**FIGURE 7:**
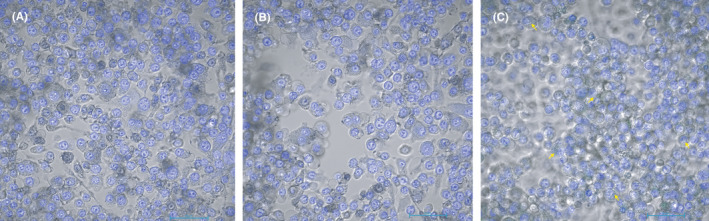
Results of confocal laser scanning imaging: the binding of NF‐κB‐p65‐targeted MBs and cells (Magnification: ×400). A: Group 1: RAW264.7 without treatment; B: Group 2: RAW264.7+naked MBs; C: Group 3: RAW264.7+ NF‐κB‐targeted MBs

The confocal laser scanning images are presented in Figure 7. For Group 1 without treatment and Group 2 treated with naked MBs, no green fluorescence was detected intracellularly. For Group 3 added with NF‐κB‐p65‐targeted MBs, gas bubbles with green fluorescence from NF‐κB‐p65‐targeted MBs were observed.

### 3.4 In vivo CEUS imaging of animal model

#### 3.4.1 Clinical assessment

**FIGURE 8:**
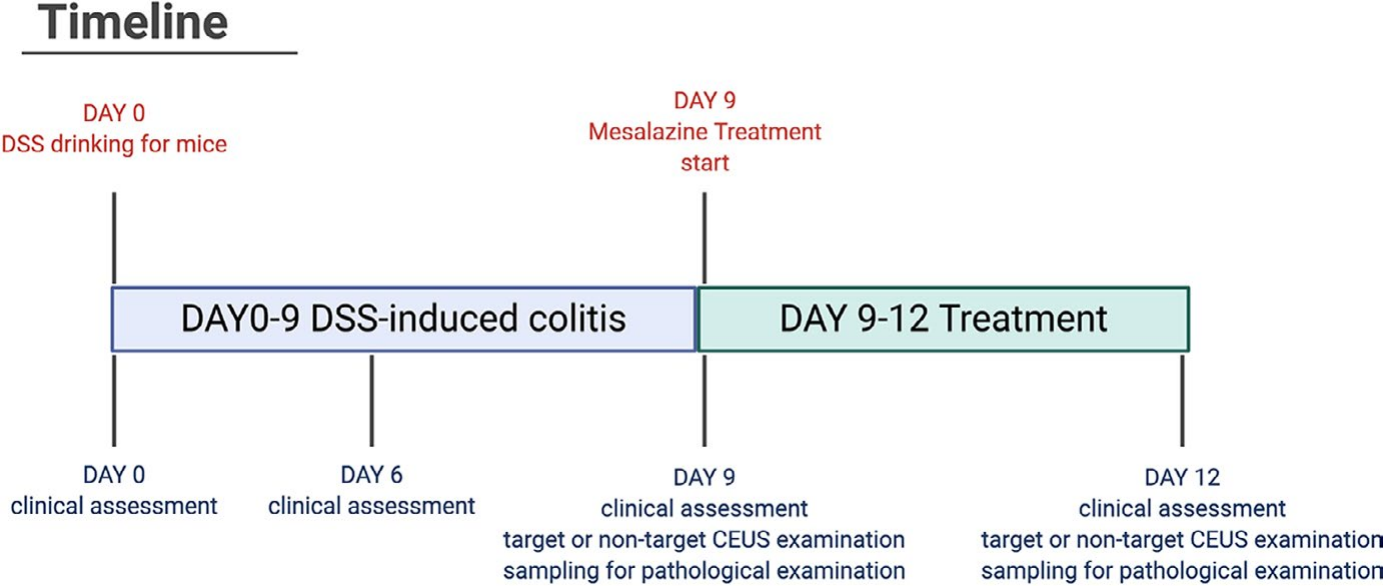
The timeline for in vivo experiment. From Day 0 to Day 9, the mice were fed with 3% dextran sodium sulphate to develop colitis, and clinical assessments were performed on Days 0, 6 and 9. On Day 9, DSS‐containing drinking water was stopped, and the experimental group was treated with Mesalazine Enemas for consecutive 3 days. CEUS was performing before treatment on Day 9 and after treatment Day 12, respectively

The timeline for in vivo experiment is presented in Figure 8. On Days 0, 6, 9 and 12, the average weight of IBD mice was 23.2 ± 0.7g, 20.1 ± 0.3g, 18.7 ± 0.3g, 19.1 ± 0.5g, respectively. In contrast, the average weight of healthy mice was 22.5 ± 0.3g, 22.9 ± 0.2g, 23.1 ± 0.4g, 23.5 ± 0.3g on Days 0, 6, 9 and 12, respectively. The weight of IBD mice on Day 0 was significantly higher than that of Days 6, 9 and 12 (***p***<0.001). On Days 6 and 9, bloody mucosanguineous faeces were observed in the IBD mice. And the stool turned to normal status after drug treatment of the mice on Day 12.

#### 3.4.2 CEUS results

**FIGURE 9:**
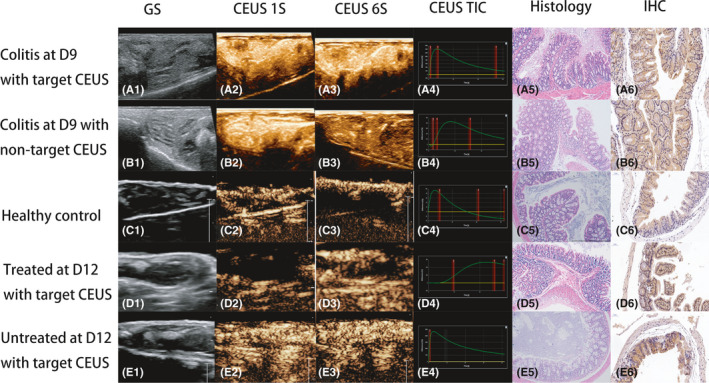
In vivo imaging and the corresponding pathological results. 1. Grey‐scale US; 2. The 1st second of CEUS; 3. The 5th second of CEUS; 4. Time‐intensity curve (TIC) of CEUS (Y‐axis: signal intensity [%], X‐axis: time [s]); 5. HE staining results of colon tissues (Magnification: ×10); 6. IHC of NF‐κB‐p65 expression of colon tissues (Magnification: ×10). A. Mice with colitis receiving target CEUS imaging using NF‐κB‐targeted MBs on Day 9. After injection, the signal intensity of intestinal wall rapidly increased (PI: 14390%). The TTP was about 6.1s. Then, the signal intensity decreased slowly, but still maintained at a high level, significantly higher than surrounding tissues (A1‐4). The inflammatory signs could be observed on HE staining (A5), and a high level of NF‐κB‐p65 expression was identified (A6). B. Mice with colitis receiving non‐target CEUS imaging using naked MBs on Day 9. Significant enhancement was presented (PI: 582%). The TTP was about 8.4s. Then, the signal intensity decreased slowly to a relatively low level, but higher than surrounding tissues (B1‐4). Inflammatory signs of the intestinal wall and enhanced NF‐κB‐p65 expression could also be detected in pathological results (B5‐6). C. Healthy controls receiving CEUS on Day 9. A rapid enhancement was observed, followed by a relatively slow subsiding. The intensity was equal to the surroundings (C1‐4). Normal bowel tissues were identified by pathology (C5‐6). D. Mice with colitis and treated with anti‐inflammatory drugs at the 12th day receiving target CEUS imaging using NF‐κB‐targeted MBs on Day 12. The signal intensity grew slowly (PI: 579%) and remained at a relatively high level with minimal decrease. The TTP was about 40.5s (D1‐4). In pathology, minimal inflammatory changes were identified after treatment (D5). The NFκB‐p65 expressions on segments of intestinal wall decreased after treatment (D6). E. Untreated mice with colitis receiving target CEUS imaging using NF‐κB‐targeted MBs on Day 12. Rapid enhancement and high level of peak intensity were obtained, similar to the results of A (E1‐4). Severe inflammation of the intestinal wall and high level of NF‐κB‐p65 expression were illustrated by pathology (E5‐6)

The schematics of CEUS imaging and TICs are presented in Figure 9, and the CEUS parameters of the five groups are listed in Table 2.

**TABLE 2:** CEUS parameters and quantitative IHC results of the five groups of mice.

	PI (%)	TTP (s)	IHC results of NF‐κB‐p65	
			p65 AOD (IOD/Area)	SD
Group A: NF‐κB‐targeted CEUS +IBD mice on Day 9	34835 (13379–73492)	7.8(5.2–13.5)	0.2748	0.0307
Group B: Non‐target CEUS +IBD mice on Day 9	437 (236–901)	6.3 (5.1–10.8)	0.2231	0.0287
Group C: NF‐κB‐targeted CEUS +healthy mice on Day 9	130 (79–231)	10.8 (9.8–13.1)	0.1230	0.0233
Group D: NF‐κB‐targeted CEUS +Mesalazine‐treated IBD mice on Day 12	528 (274–779)	35.7 (18.1–49.5)	0.0874	0.0237
Group E: NF‐κB‐targeted CEUS +untreated IBD mice on Day 12	39957 (15493–63405)	8.3 (4.2–12.5)	0.2453	0.0354

AOD: Average optical density; CEUS: contrast‐enhanced ultrasound; IBD: inflammatory bowel disease; IHC: Immunohistochemical; IOD: Integrated optical density.

Group A: After injection of MB bolus, significant enhancement of the hypertrophic colon wall was observed, followed by a slow subsiding, and the enhancement was kept at a high level (Figure 9A1‐4).

Group B: Significant enhancement and slow subsiding were also observed after injection. The contrast signals were decreased to a relatively low level at the end of the imaging process, but higher than surrounding tissues (Figure 9B1‐4).

Group C: Significant enhancement and fast subsiding were presented, and the peak intensity was equivalent with surrounding tissues. After subsiding, the signal intensity was lower than the surroundings (Figure 9C1‐4).

Group D: Slow increment of signals was visualized, and the peak intensity was slightly higher than surrounding tissues. The enhancement remained for a relatively long period and decreased slowly (Figure 9D1‐4).

Group E: The enhancement pattern was similar to Group 1 (Figure 9E1‐4).

The PI of Group A (target CEUS of IBD mice with colitis on Day 9) was significantly higher than Group B (non‐target CEUS of IBD mice), Group C (healthy controls) and Group D (target CEUS of treated IBD mice on Day 12) (mean value of PI: 34835%[13379–73492%] VS 437%[236–901%], 130%[79–231%], 528%[274–779%], ***p***<0.0001 for all of the comparisons). The TTP of Group D was significantly higher than Group A‐C and E (mean value of TTP: 35.7s [18.1–49.5s] VS 7.8s [5.2–13.5s], 6.3s [5.1–10.8s], 10.8s [9.8–13.1s], 8.3s [4.2–12.5s], ***p***<0.0001 for all of the comparisons).

#### 3.4.3 Histological and IHC results

The HE and IHC staining results are presented in Figure 9, and quantitative IHC results of NF‐κB‐p65 expressions are shown in Table 2. On Day 9 after DSS drinking, the intestinal villi became shorter, and the submucosa was highly oedema. The necrosis and shedding of intestinal epithelial cells and lamina propria cells could be seen, and a large number of inflammatory cells infiltrated. For the control group, HE staining results showed normal intestinal villi and occasional inflammatory cell infiltration. After treatment, mucosal oedema of the intestinal wall of the mouse was not obvious, and necrotic exfoliated cells were seen in some segments, and infiltration of a small number of inflammatory cells was also observed.

On Day 9, p65 staining increased significantly compared with the control group (mean value of AOD: 0.2748 VS 0.0735, *p*<0.01). Significant decrease of p65 staining was demonstrated after treatment on Day 12 (mean value of AOD: 0.0874, *p*<0.01).

## 4 DISCUSSION

In this study, NF‐κB‐targeted MBs were fabricated by connecting NF‐κB‐p65 Abs onto the surface of MBs, and the MBs were proved be specifically and effectively conjugated with the inflammatory cells in vitro with high expressions of NF‐κB‐p65. Targeted CEUS was performed on DSS‐induced IBD mice, and a pattern of fast enhancement and slow subsiding was observed in target CEUS group of IBD mice. The peak intensity was significantly stronger than using naked MBs. After treatment with anti‐inflammatory drugs, the slow enhancement pattern was presented, and the peak intensity also significantly decreased, in accordance with pathological and IHC results of NF‐κB‐p65 expressions. The results indicated that target CEUS could accurately reflect the changes of NF‐κB‐p65 expressions on inflammatory colon tissues during the disease course. It is feasible to utilize NF‐κB‐targeted CEUS in diagnosing and evaluating intestinal inflammation of IBD.

NF‐κB is one of the most important cytokines expressed at high levels in IBD and plays an essential role in the mechanism of inflammatory damages of the disease, such as local hypoxia and chronic fibrosis, and mucosal healing.^22^, ^23^, ^24^, ^25^ NF‐κB could act as a key target for some newly developed therapeutic agents of IBD.^26^, ^27^, ^28^ It has also been validated by previous researches that the cytokine is intensively involved in the development of CAC. A sustained high level of NF‐κB of the intestinal epithelial cells can escalate the risk of CAC.^29^ On the other hand, some therapeutic agents can suppress tumorigenesis by acting on NF‐κB signalling.^13^, ^14^ Consequently, it can be helpful in evaluating inflammatory activity and monitoring treatment efficacy of IBD by detecting NF‐κB expressions on intestinal walls. Yang et al. used bioluminescence imaging to trace the NF‐κB gene expressions of IBD mice in vivo.^30^ While, bioluminescence imaging is generally used for research experiments, and its clinical utilization is limited. A clinically applicable imaging method for molecular imaging should be developed to meet the demand.

Target CEUS, enabling quantitative characterization of the target molecules in vivo, can provide a novel way for precise management of the disease. In this study, the changes of NF‐κB‐p65 expressions of the disease course, from occurrence of colitis to reduced inflammation activity, could be accurately identified by target CEUS, and the signal intensity within the lesions was much higher than conventional CEUS, implying the high binding rate of target MBs and local tissues. Besides, the high‐signal intensity presented in the target CEUS of inflammatory bowels might imply a high sensitivity for target CEUS in detecting early inflammation and reflecting treatment efficacy, and further studies are necessary to reveal the value of target CEUS in identifying the early process of IBD.

Bowel ultrasound has been popularized worldwide currently, as an important imaging method for evaluating IBD. Apart from its high cost performance, the accuracy of high‐frequency US in detecting inflammatory lesions on the intestinal wall has been recognized by clinicians.^31^, ^32^ CEUS has also become a mature imaging technique and has been applied in a variety of diseases.^33^ Based on conventional US and CEUS, target CEUS can be acceptable for both doctors and patients, and easy for clinical promotion. Meanwhile, ultrasonic MBs are safe imaging agents with almost no side effects and allergic responses, which also facilitates the utilization of target MBs. Moreover, some target treatment strategies, such as delivering anti‐inflammatory drugs and nucleic acids, could also be performed through the use of target MBs.^34^ And we aim to design therapeutic MBs that load therapeutic drugs and target molecules to perform molecular imaging‐guided target therapy for IBD in further study. By paving the way for in vivo molecular imaging of NF‐κB, we believe that target CEUS can possess great clinical potentials for IBD.

To note, NF‐κB‐p65 is a category of nucleic proteins. The combination of targeted MBs and RAW267.4 cells could be identified through confocal laser scanning, but we could not verify the binding of MBs to the cellular nucleic, because the MBs could not enter the cellular membranes. On the contrary, a high rate of combination between the NF‐κB‐targeted MBs and the inflammatory bowels was identified by CEUS in our study. This could be explained by the intracellular and extracellular distributions of the cytokines, which might be caused by the altered cell membrane permeability and local cell necrosis within the inflammatory tissues. And in further study, we plan to explore the specific binding locations of the NF‐κB‐targeted MBs in the inflammatory tissues of IBD bowels.

There exist several limitations in the study. In this study, we did not perform the longitudinal evaluation of NF‐κB‐p65 expressions in the whole process of genesis and development of inflammation. To evaluate the sensitivity of target CEUS in identifying the cytokines, more studies in the early phase of inflammation should be designed. And as imaging methods have been attached with great importance in disease monitoring of IBD, target CEUS may also possess clinical potential in the surveillance of drug efficacy and relapse of inflammation, which requires further studies for verification. Therefore, more researches should be designed for investigating the role of the NF‐κB‐targeted MBs in treatment monitoring in the future.

In summary, we fabricated the NF‐κB‐targeted MBs and validated the specific binding of the MBs and inflammatory cells. The feasibility of NF‐κB‐targeted CEUS in detecting and quantifying NF‐κB‐p65 expressions was proved on IBD animal model in vivo. Further clinical utilization of the NF‐κB‐target CEUS can be expected.

## CONFLICT OF INTEREST

The authors declare no conflict of interest.

## AUTHOR CONTRIBUTION

**Chenyang Zhao:** Conceptualization (equal); Data curation (lead); Formal analysis (equal); Methodology (equal); Project administration (lead); Software (equal); Visualization (equal); Writing‐original draft (lead). **Li Ma:** Conceptualization (equal); Data curation (equal); Formal analysis (equal); Software (equal); Visualization (equal); Writing‐review & editing (equal). **Yanwen Luo:** Formal analysis (equal); Investigation (equal); Project administration (equal); Software (equal); Validation (equal). **Wenbo Li:** Conceptualization (equal); Formal analysis (equal); Methodology (equal); Resources (equal); Software (equal); Supervision (equal). **Mengsu Xiao:** Conceptualization (equal); Data curation (equal); Methodology (equal); Resources (equal); Supervision (equal); Writing‐review & editing (equal). **Qingli Zhu:** Conceptualization (lead); Data curation (equal); Formal analysis (equal); Funding acquisition (equal); Methodology (equal); Resources (equal); Software (equal); Supervision (equal); Validation (equal); Writing‐review & editing (lead). **Yuxin Jiang:** Conceptualization (equal); Formal analysis (equal); Funding acquisition (equal); Methodology (equal); Resources (equal); Supervision (equal).

## Data Availability

The data that support the findings of this study are available from the corresponding author upon reasonable request.
